# A case study in teaching and learning the design, conduction, and analysis of Bayesian adaptive trials through an application

**DOI:** 10.1017/cts.2026.10796

**Published:** 2026-07-14

**Authors:** Naima Alam, Alexandra Brown, Xiaosong Shi, Sreejata Dutta, Kate Young, Jianzheng Wu, Kaustubh Nimkar, Geethanjalee Mudunkotuwa, Elena Shergina, Joshuna Bernal, Md Tamzid Islam, Lauren Yoksh, Junqiang Dai, Nasrin Sultana, Milind A. Phadnis, Jo Wick, Byron Gajewski

**Affiliations:** 1 Biostatistics & Data Science, University of Kansas Medical Center, Kansas City, KS, USA; 2 ACCORDS (Adult and Child Center for Outcomes Research and Delivery Science), University of Colorado School of Medicine, Aurora, CO, USA

**Keywords:** Case study, learning experience, adaptive randomization, student engagement, trial design

## Abstract

**Introduction:**

Gaining clinical trial design and management experience can be challenging for students in class. Opportunities for students to participate in designing and implementing a “from scratch” design are essential to succeeding after graduating. Limited materials are available that provide meaningful opportunities for learners to practice and implement adaptive trial design. As a solution, we developed an open-source case study framework that instructors can use directly in a course to give students hands-on experience with Bayesian adaptive trial design and implementation.

**Materials and methods:**

We developed an open case study (DRIVE trial) project, which offers an instructor-ready framework organized into seven components. Each component is designed to map onto a distinct stage of a clinical trial and include materials that instructors can adapt to match their course level and chosen scientific questions. These components are tied together by a worked example in which, after comparing multiple study designs, a Bayesian adaptive design with response adaptive randomization was chosen and implemented throughout the framework.

**Results and discussion:**

The framework provides instructors with a complete, ready-to-use template for bringing adaptive trial experience into the classroom without building a case study from scratch. The study’s significance lies in its broader implications for biostatistics student training and experience. The trial underscored the effectiveness of comparing study designs and the necessity for collaboration when implementing a clinical trial. This offers valuable practical training and resources for future biostatisticians, serving as a template across various fields. Supplementary materials for this article can be found online (https://github.com/BayesPhase/DRIVE).

## Introduction

A major challenge in the practice of teaching graduate Biostatistics students is the limited number of available course materials that provide meaningful opportunities for students to engage in the design, implementation, and analysis of Bayesian adaptive clinical trials within a reasonable timeframe. Furthermore, studies have suggested that students’ engagement in hands-on activities, simulation, and discussion is necessary to develop deeper knowledge in clinical trial design and implementation [1]. To address this problem, we developed an open-source, case-study-based framework, the Daily Route Investigation Via an Effective (DRIVE) Bayesian adaptive trial project, that instructors can use directly in a course or workshop to provide students with hands-on experience in Bayesian adaptive trial design and execution. The primary purpose of the paper is not to report the results of the DRIVE trial, but to describe the framework, explain how each component works, and provide clear guidance on how an instructor can adapt these materials for their own course of study.

The importance of Bayesian adaptive designs has been discussed widely in the literature [2–7]. Adaptive designs in clinical trials have increased dramatically over the past decade due to their flexibility and potential to assign more participants to the treatment arm with superior outcomes [8,9]. Specifically, response-adaptive randomization (RAR) is a popular adaptive design feature that involves assigning more participants to better-performing arms during a trial, making the trial more efficient [6,10–14]. Many papers have been published on RAR that explained the study design, but not the details of the experience of conducting it. More literature is needed on providing students with an active learning environment to increase their experience with these trial designs [15,16]. The goal of this paper is to fill that gap.

The DRIVE trial exemplifies a framework organized into seven components. This resource contains in-depth, self-contained, and engaging (including different design arguments, protocol development, data management plan, simulation ideas, and analyses) components that demonstrate illustrative data analyses covering a diverse range of Bayesian adaptive trial designs to teach learners how to effectively design a clinical trial, derive knowledge from data, and implement. Each component is designed to map onto a distinct stage of a real clinical trial, so that students work through the full pipeline rather than isolated pieces. An instructor can choose to show the solutions provided in the case study or leave a discussion open-ended. The goal is flexible; the educator can simply customize the study by removing or expanding components to match their course level difficulty, time constraints, and student engagement. While the DRIVE trial is not a medical application, the idea can be extended to medical applications such as optimizing sleep and dietary intake, which we discuss. This guide can be used by instructors to bring applications to the forefront in the classroom, or it can be used by independent learners outside of the classroom.

All the materials, including the full protocol template, statistical analysis plan (SAP), simulation code in R etc., are available online at the project repository (https://github.com/BayesPhase/DRIVE). Our reasoning for providing a full-length guide is that it is typically easier for an instructor to remove or modify materials instead of creating it from scratch when designing a Bayesian adaptive clinical trial. In addition, this method is particularly helpful for instructors who may not feel confident creating a design from scratch, specifically if it’s outside their main area of expertise, as our case study is built with domain experts. In that way, we aim to reach a broader audience than just instructors in a classroom, as any learner interested in a particular topic can walk through the case study to see an example of how to design and implement a trial and conduct a complete data analysis.

## Framework overview

A working group was created through the Department of Biostatistics & Data Science at our university in 2018 with a focus on designing more efficient trials utilizing FACTS^TM^ (Fixed and Adaptive Clinical Trial Simulator) software [17]. FACTS is a powerful tool developed by Berry Consultants that allows biostatisticians to quickly design and compare both fixed and adaptive trial designs through simulations to optimize study designs. Anyone not familiar with the FACTS terminology and interested in learning, can look up the documentation available online for understanding and using the software [18]. Previously, the working group focused on optimizing trial designs with varying parameters but had yet to be equipped with implementation and final analysis experience. As many of the members are future biostatisticians, the group felt conducting a case study from start to finish would give experience in executing, monitoring, analyzing, and interpreting results for a clinical trial. Specifically, this study gave us a platform to experiment with sophisticated and novel features of clinical trial designs such as response adaptive randomization and early stopping criteria [19–21].

### Structure of the seven components

The framework is organized into seven main components, each corresponding to a distinct stage of a clinical trial. Table 1 below provides the instructor with a roadmap summarizing each module.

**Table 1.** Overview of the seven components Table 1 long description.

Components	Purpose
1. Proposed idea	Define scientific question, treatment arms, and primary endpoint.
2. Design selection	Compare different design ideas through simulation and select optimal design
3. Protocol	Translate the selected design into a formal trial protocol.
4. SAP	Defining the model, interim analysis rules, and decision thresholds.
5. Data management plan and determination of non-human subjects	Details of how data were collected, stored, and protected.
6. REDCAP form	Details on the RedCap form used for the trial to build the data collection instrument.
7. R code	Implement the analysis and interpret the results.

SAP = statistical analysis plan; REDCap = Research Electronic Data Capture.

The components are designed to be taught in sequence as each is built on decisions made in the previous stage. However, instructors can modify materials due to course time constraints. For example, in a one-day workshop focused on Bayesian analysis, the instructor could hand students a complete protocol and SAP and begin with component seven directly.

### How to adapt the framework

**Figure 1.**
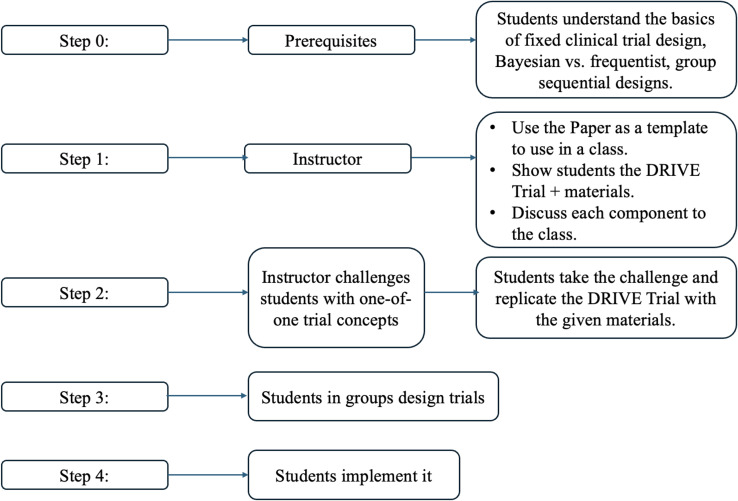
A step-by-step guide for instructors to adapt the framework in a classroom setting. DRIVE = daily route investigation via an effective (DRIVE) Bayesian adaptive trial.

The DRIVE trial case study uses drive time as the primary outcome, as it is logistically simple, endpoint is immediate, and does not require Institutional Review Board (IRB) approval for human subjects. It is designed to illustrate the use of adaptive trial design without the regulatory and ethical concerns of a medical study. We want to highlight that while the structure of the case-study may be similar to others [22–24], our case study has a different purpose. The example reference papers provide case studies that are already implemented. However, our case study is a prospective implementation of the trial. The workflow below (Figure 1) serves as a guide for an instructor to adapt the framework in a classroom setting.

To use these materials effectively, first, students should have prior exposure to the basics of fixed clinical trials, Bayesian vs frequentist methods, group sequential design and statistical computing. Instructors should review these topics during a course before introducing the case study. The target audience includes advanced graduate-level biostatistics, data science or public health students. However, the case study is also adaptable for advanced undergraduate students or learners in related fields (e.g., medicine, epidemiology) who seek to have hands-on experience with the concept of adaptive trial design.

An instructor can use the materials to provide a framework for designing a future trial that they could lead in their course. Some relevant ideas include sleep study, coffee intake, hypertension management, weight loss study etc. Studies on hypertension or weight loss may require more time and clinical oversight. A simple example could be a sleep study comparing different bed angles (high, medium, flat) to find whether an adjustable bed is worth buying for better sleep. The class projects should be considered as non-human subject research, however having students go through the IRB is valuable to learn the process of submitting a protocol. Instructors should calibrate the depth of each component to their available course time, rather than treating the DRIVE trial implementation as a fixed target to replicate exactly.

## Component by component instructor guide

We will describe the seven individual components of our case study using the DRIVE trial as an example. This case study showcases how to design a trial, implement it and use FACTS and R statistical programming language for data analysis.

### Proposed idea

The first section provides a guide to define the scientific question, covering the study population, treatment arms, primary endpoint, etc., for a trial. The instructor should open this section by presenting example study ideas and leading a discussion about what makes a question suitable for a clinical trial. If students propose their own ideas rather than using the DRIVE framework, the instructor should set clear feasible criteria such as requiring finishing the trial within the semester, not requiring full IRB review etc. In the proposed DRIVE trial, the aim was to find the optimal driving route from a participant’s home to the workplace among three candidate routes with one subject driving each workday. Each drive served as one experimental unit. We labeled these routes as Rainbow, State Line, and Plaza/Control. The Plaza route was the slowest expected compared to the other two routes and thus was established as Control. The primary aim of this case study was to select the route (out of two comparator routes) that had a higher probability of being faster than the Control arm (Plaza route).

Once the study idea is established, students should be challenged to optimize the trial design in terms of operating characteristics and different design feature ideas. In the DRIVE trial, the working group was presented with the challenge to optimize the trial design in terms of the operating characteristics, such as power, expected total sample size needed, and expected trial duration. Multiple trial design features were considered, such as varying statistical models, RAR, as well as early and final stopping rules. To optimize the study design, the members of the working group were broken into five small groups and were given time to work on their own designs and then report back to the working group. The following criteria were set with the challenge of designing the most efficient trial:

- The maximum number of drives included in the study was 33. This was determined from a fixed design calculation for a three-arm trial using Bonferroni adjusted alpha with 90% power for a two-sided comparison of the mean drive time between routes [25].
- Three treatment response scenarios were proposed: null hypothesis (all routes have the same drive time), expected (per Google Maps), and one best. These are summarized in Table 2.
- To compare two driving routes to a single Control route, the Bonferroni correction needs to be used to adjust the type I error rate from 0.05 to 0.05/2 = 0.025 [26].

**Table 2.** Three proposed treatment response scenarios Table 2 long description.

	Drive time (minutes)		
Scenario	Plaza (control)	Rainbow	State line
Null	15	15	15
Expected	15	13	13
One best	15	13	14

The standard deviation was 1.2 for each route.

At least one minute difference in drive time was considered clinically meaningful. The fixed standard deviation was based on preliminary data and assumed constant across routes for simplicity, allowing focus on the Bayesian adaptive features rather than variance modeling.

### Design selection

This component is the core of the framework, which asks students to compare multiple trial designs by simulating and evaluating through operating characteristics. Before students begin, the instructor should introduce RAR, early stopping rules, and the decision threshold so that students have a principled basis for comparison. If FACTS is available, the instructor can demonstrate it shortly; if not, the provided R simulation code should be utilized before students work with it in groups to design a trial.

In the DRIVE trial, to evaluate operating characteristics simulation was conducted under three different scenarios shown in Table 2. For each simulated trial, drive times were generated randomly from a normal distribution with the true mean and standard deviation under each scenario. After each drive, a Bayesian update was performed to evaluate the posterior probability that each route was better than Control. The process continued until a decision threshold met or total drive was completed. This process was repeated around thousands of times to estimate how often it picked the true best route compared to the control. For teaching purpose, the exercise can be simplified to a fixed design and use this as a power calculation using closed form under the traditional frequentist framework [25]. In the supplement materials in the GitHub repository, we provided sample code for this alternative design so that students who have no experience with the Bayesian approach or simulation can learn the design implementation without much difficulty.

Several study designs were proposed by the student groups for this trial, including one fixed and four adaptive designs [6,27]. For example, for the early success or futility rule, see reference 5 from a regulatory point of view. For response adaptive randomization, a specific example can be found in reference 27. All the design details can be found in the GitHub repository in the supplement materials.

The PI was responsible for choosing the best design among all five designs. Each design was evaluated in terms of its ability to control the overall type I error rate, power to detect differences in the travel times of the routes being assessed, the expected sample size (number of drives), and feasibility in terms of the number of interim analyses needed. The route to compare to the Control was picked based on the maximum posterior probability of Rainbow or State Line having the lowest drive time. The power and expected sample size for each study design and simulated virtual trials are summarized in Table 3.

**Table 3.** Proposed designs by working group to compare Table 3 long description.

Design type	Number of interims (at *N* drives)^1^	Early stopping criteria	Final criteria	Scenario (effect sizes)	Mean *N* drives	Type 1 error rate/ power
Fixed	0	None	Success	Null	33.0	0.025
				Expected	33.0	0.978
				Best	33.0	0.934
RAR, lower interims	3 (**8**, 16, 24)	Futility and Success	Success	Null	23.6	0.024
				Expected	23.6	0.980
				Best	25.1	0.944
RAR, arm dropping	1 (**15**)	Futility	Futility and Success	Null	30.5	0.023
				Expected	32.9	0.987
				Best	32.9	0.932
RAR, higher interims	5 (**10**,15,20,25,30)	Futility and Success	Success	Null	25.6	0.020
				Expected	22.5	0.978
				Best	24.1	0.935
RAR, no fixed allocation to Control	5 (**10**,15,20,25,30)	Futility and Success	Success	Null	25.7	0.026
				Expected	24.3	0.902
				Best	25.4	0.886

^1^All bolded *N* values represent when we start to evaluate the early stopping rules. *N* = number of drives, RAR = response adaptive randomization.

We can briefly summarize the difference between the four adaptive study designs by a few features: the frequency and duration of interim analyses, and whether the design incorporated a fixed allocation to the Control arm throughout the study. Under no fixed allocation to control, all three routes were considered for RAR [27]. We compared the overall performance, in terms of mean expected number of drives, and type I error rate/power across the simulations for the designs presented in the working group. All had appropriate type I error rates (controlled at around 0.025) for the null, and adequate power (>80%) to detect a better drive time compared to Control for the comparator routes. Compared to the fixed design, almost all adaptive designs saved drives needed for all treatment scenarios. After discussing each design’s pros and cons, the designated principal investigator (PI) chose a Bayesian adaptive design using RAR and fewer interim analyses (RAR, lower interims, Table 3) with comparable power and type I error rates. The adaptive features allowed for the potential for fewer drives and higher power compared to a more traditional fixed design [28–31]. With this design, there were a lot of early sample savings and very competitive operating characteristics with only three interim analyses. Having fewer interims made the overall process more efficient as the logistic burden was decreased. These features made the design more efficient compared to others.

### Protocol

The protocol is the formal written plan that shows how the trial will be conducted. This component asks students to translate their design decisions from component 2 into a complete document. The instructor should provide the DRIVE trial protocol to the students and emphasize it before the students use it. In the DRIVE example, Students in a team wrote the protocol prior to the main trial, which is a crucial part of clinical trial designs. Writing a protocol helped students understand and find common challenges researchers can face while planning and running a trial [32]. A mock trial was conducted before starting the main trial to work out all the logistics and any challenges the study team might face during the actual trial [33]. The team members had an opportunity to practice effective communication and learned how to solve challenges that appeared during the trial’s conduct. During the trial, email updates were sent to the working group to keep everyone on track. Frequent emails ensured a clear and effective communication and kept students informed about the study [34]. After the first interim analysis, a discussion session was held to review what went well and what difficulties students faced to improve the processes for the next interim.

### Statistical analysis plan

The SAP provides details on how the trial data will be analyzed, covering statistical modeling, prior distributions, decision criteria, interim, and final analysis. The instructor should introduce the Bayesian modeling concepts, explaining posterior distribution, posterior probabilities, and adaptive decisions. In the DRIVE trial, the primary endpoint was drive time for a day, modeled as a continuous variable measured from key-in to key-out. A Bayesian approach was utilized to determine adaptive decisions by considering posterior probabilities. The posterior was evaluated using Markov chain Monte Carlo (MCMC) with individual parameters updated by Gibbs sampling (or Metropolis-Hastings where possible) [35–37], using only the outcome data available at the time of the analysis. Although the posteriors could be obtained analytically, the use of MCMC guides students to understand the computational intensity under Bayesian modeling that is commonly used in adaptive trial simulation studies. The Bayesian framework allows for the calculation of posterior probabilities that a comparator route is faster than the Control, facilitating adaptive randomization and interim decision-making. The decision threshold, including success and futility, was chosen to balance the type I error at 0.025 while ensuring acceptable power (>80%). This was done by a trial-and-error process to balance between power and type I error.

For the chosen design, we simulated 1000 virtual trials, tracked each trial’s outcome, and aggregated them to calculate the operating characteristics, including the expected number of drives, the mean drive time by route, expected allocation in each route, probability of success, probability of futility, and expected duration. For each simulated trial, we considered 2500 MCMC draws with 1000 burn-ins. The results from all simulation trials for each scenario, along with the detailed analysis plan, can be found in the supplementary materials.

The final design required one subject to conduct drives on weekdays only, with inclusion criteria that required drive times to start between 7:00 AM and 8:30 AM, excluding extreme weather, weekends, and campus closures. Any incomplete drives would be considered dropouts. The trial began with a burn-in allocation ratio of 2:1:1 for Plaza/Control, Rainbow, and State Line, respectively. After the first interim analysis, the subsequent 8 drives were assigned based on the adaptive allocation rule. The subject was blinded to the randomized route until just before departure to prevent any perception bias in driving performance. In the context of this study, complete blinding was not possible as the study subject inevitably became aware of the route once the drive began. The route disclosure was delayed until departure to avoid any pre-drive expectations. Again, the potential for post-disclosure bias influencing the outcome was minimal. All the statisticians (except the PI and the data analysts) decided to be unblinded. Any AEs would be self-reported by the subject through a survey following each drive. These AEs include problems related to road or vehicle conditions, or any issue leading to a significant delay or route change.

### Data management plan and determination of non-human subjects

This component illustrates how data will be collected, stored, and protected throughout the trial. The instructor can follow the DRIVE example to describe how data should be managed. The DRIVE trial project involves giving biostatisticians experience in designing, conducting, and analyzing a Bayesian adaptive clinical trial that uses applicable commute data points to establish the most effective driving route for a study subject. Ethical dimensions are an important concern in clinical trials involving human subjects; the DRIVE trial is suitable for the determination of non-human subjects. This project is for educational purposes, and the driver was the only study participant whose data would be collected from, with no personal health information being collected. A “non-human subjects” determination was made by our own institution’s IRB. The study was focused on learning how to implement adaptive designs in real life without the regulatory and ethical constraints of human subject research. As determined by the university’s Institutional Review Board (IRB), the study did not involve human subjects and did not require a full IRB review. The randomization for the initial interim was executed by the data managers using R version 4.3.0, utilizing the R package “randomizeR” [38]. The randomization list for the subsequent interims and final analysis phase was generated using the “Analysis” tab of the FACTS software, based on the data collection in all the preceding interims. Data were managed and analyzed by the study team, ensuring the blinding and integrity of the analysis. As per protocol, at first, the PI and data analysts were blinded among the study team. After the first interim, the analysts were unblinded as blinding requires multiple rounds of code translations, which increases the risks of making errors, and unblinding to the analyst would not introduce bias in the analysis. The PI remained blinded to ensure there was no perception bias. The data safety monitoring board, composed of two experienced biostatistics faculty, played a pivotal role in evaluating reports and making decisions regarding the continuation or early stopping of the trial.

### REDCap form

In the DRIVE trial, after each drive, the subject completed an online survey administered on our university’s Research Electronic Data Capture (REDCap) [39,40] (see REDCap form in supplementary materials). The survey was designed by the study team to gather all the information for the calculation of the mean drive time. Regarding any app outage, as a backup plan, someone from the data manager group would accept a call from the subject to hear their response to the route that day. The study design ensured that the subject’s participation did not expose them to undue risk, and all data collection activities were scheduled post-commute to avoid distractions. An instructor can provide students with the example REDCap form for illustrative purposes. If REDCap is not available, Google Forms or other ways can be substituted, although students should be made aware that REDCap is the standard platform in clinical research.

### R code

The final section covers the actual implementation of the Bayesian analysis, bringing together all preceding components. This section includes information on the R code [41] used to analyze the data. The instructor will give a brief overview of the code; however, students are expected to understand how R code works and should be able to modify the code. All the reports and simulations were run using R version 4.3.0 [42] and FACTS^TM^ (Berry Consultants, LLC, Austin, TX) version 7.0.0. Academic and industrial licenses can be obtained from Berry Consultants. However, the team replicated the FACTS simulations in R so that readers without the proprietary software could replicate them (see supplementary materials).

In the next section, we describe the trial implementation in detail so that an instructor can use it to compare it with what the students will do. We described how the trial was executed, the interim analyses, and the final analysis results.

## Implementing the trial

### Trial execution

**Figure 2.**
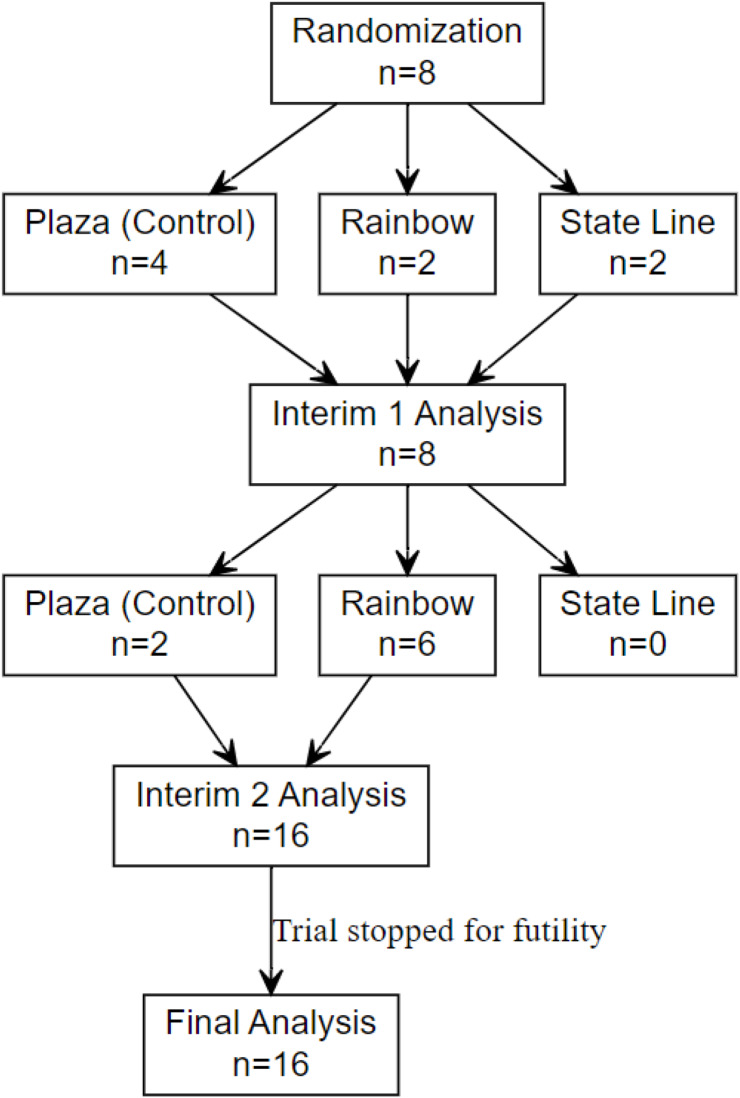
Consort diagram.

The DRIVE trial was planned from January 16, 2024, to February 29, 2024, and implemented from January 16, 2024, to February 12, 2024. The CONSORT (Consolidated Standards of Reporting Trials) diagram illustrates the actual trial flow (Figure 2). To begin the randomization of 8 drives, four routes were allocated to Plaza/Control, two to Rainbow, and two to State Line. Following the completion of these initial drives, the first interim analysis was conducted. Based on this interim analysis, the allocation was adjusted for the next 8 drives, where Plaza/Control had two drives, as it was a fixed Control allocation, and Rainbow had an increased allocation of 6 drives. In contrast, State Line did not receive additional allocation due to low allocation probability. After the 16 drives, a second interim analysis was conducted, which resulted in early stopping for futility, suggesting that the comparator routes did not demonstrate a significant improvement over the Control. Therefore, the final analysis was conducted with these 16 drives.

In parallel to the route allocation, Table 4 illustrates several factors that may affect drive time by randomized route. The table highlights varying weather conditions across the routes, with Plaza/Control encountering mixed conditions, Rainbow having primarily clear conditions, and State Line facing icy conditions. Additionally, the start time allows us to evaluate the route consistency under different traffic flows. Most of the time, the drives were started between 7-8 AM. Regarding drive days of the week, as per participants’ experience, Wednesdays and Thursdays tend to be the highest in terms of congestion. Typically, Fridays were a little lighter in traffic than the other days.

**Table 4.** Summary of factors that may affect drive time Table 4 long description.

	Route randomized, *n* (%)		
	Plaza (control) *N* = 6	State line *N* = 2	Rainbow *N* = 8
**Weather**			
Clear	2 (33%)	0 (0%)	7 (88%)
Icy	3 (50%)	2 (100%)	1 (13%)
Wet	1 (17%)	0 (0%)	0 (0%)
**Car type**			
GMC	6 (100%)	2 (100%)	8 (100%)
**Construction**	0 (0%)	0 (0%)	0 (0%)
**Start time (hour)**			
7–8 AM	5 (83%)	2 (100%)	4 (50%)
8–9 AM	0 (0%)	0 (0%)	4 (50%)
After 9 AM	1 (17%)	0 (0%)	0 (0%)
Days of Week*			
Monday	1 (17%)	0 (0%)	2 (25%)
Tuesday	1 (17%)	1 (50%)	2 (25%)
Wednesday	1 (17%)	1 (50%)	1 (13%)
Thursday	1 (17%)	0 (0%)	2 (25%)
Friday	2 (33%)	0 (0%)	1 (13%)

*Proportion of days: Monday (19%), Tuesday (25%), Wednesday (19%), Thursday (19%), Friday (19%).

### Interim analyses

The first interim analysis provided early insight into the comparative efficacy of the routes taken. Bayesian estimates and credible intervals (CrIs) were used as a measure of uncertainty for the parameter of interest [43]. After the first 8 drives, preliminary data suggested that the Plaza/Control route had a mean time of 11.7 minutes (95% CrI: 10.5, 13.0). Rainbow exhibited an average drive time higher than the Control, at 11.9 minutes (95% CrI: 10.3, 13.7). A slightly higher mean suggests a potential for increased travel time on this route. State Line, conversely, showed a notably higher average drive time of 14.0 minutes (95% CrI: 12.3, 15.7), suggesting greater variability and less precision in estimating mean drive time. Also, the State Line route did not demonstrate a clear advantage over the Control. The posterior probability of Rainbow being the best route was high at 0.95. Details of the modeling can be found in the SAP. In terms of decision-making criteria, the probability of being better than Plaza/Control for the best route (i.e., Rainbow) was not greater than 0.998, thus there was no early stopping for success. Moreover, the maximum probability of being better than Plaza/Control by one minute was not less than 0.1, so we also had no futility criteria. Therefore, the data did not justify stopping early for either success or futility. This led to an adaptive adjustment in route allocation, favoring the Rainbow route. The updated allocation consisted of two routes for Plaza/Control and six routes for Rainbow for the next 8 drives.

Two protocol violations were identified during the first interim. One drive started after the inclusion window (7 AM to 8:30 AM). The second violation resulted from unblinding the study team, excluding the PI, while analyzing the first interim due to difficulties the analysis team faced regarding the risk of mislabeling the routes arising from blinded data.

The second interim analysis was conducted after 16 drives. Similar to the first interim’s result, the subject spent the lowest time driving on Plaza/Control. The probability of either Rainbow or State Line being faster than Plaza/Control was low, leading to the trial being stopped for futility. Because the endpoint was immediate, no waiting was required for outcomes to be collected. Hence, the second interim and the final analysis were completed using the same data. The interim analyses tables can be found in the supplement files.

### Final analysis

As the trial stopped early for futility, a total of 16 drives were analyzed to determine the most efficient route. Table 5 shows that the mean drive time for Plaza/Control was 11.4 minutes (95% CrI: 10.4, 12.4). The mean drive time for Rainbow was slightly longer than Plaza/Control at 12.0 minutes (95% CrI: 11.1, 12.9). State Line showed a longer mean drive time (mean at 14.0 minutes, 95% CrI: 12.3, 15.7), which indicated greater variability compared to Plaza/Control. One can say State Line performed the worst among the three routes. The final analysis confirmed the trends observed during the interim analyses, confirming the futility criteria.

**Table 5.** Final analysis Table 5 long description.

Parameter	Description	Estimate (95% CrI)
*N*	Number of drives	16
*θ*_0_	Mean time for Plaza (Control)	11.4 (10.4, 12.4)
*θ*_1_	Mean time for Rainbow	12.0 (11.1, 12.9)
*θ*_2_	Mean time for State Line	14.0 (12.3, 15.7)
*σ*	Standard deviation for drive time	1.2 (0.8, 1.8)
*θ*_1_ − *θ*_0_	Mean difference between Rainbow and Plaza (Control)	0.6 (-0.7, 2.0)
*θ*_2_ − *θ*_0_	Mean difference between State Line and Plaza (Control)	2.6 (0.6, 4.6)
Pr(Max) for rainbow	Probability of Rainbow being the best route	0.98
Pr(Max) for state line	Probability of Stateline being the best route	0.02
*Pr*(*θ*_*d*_<*θ*_0_) for *d* = greatest *Pr*(*Max*)	Probability of being better than Plaza (Control) for the best route	0.16
Max *Pr*(*θ*_*d*_−*θ*_0_<−1)	Maximum probability of being better than Plaza (Control) by one minute	0.01
*Pr*(*θ*_*d*_<*θ*_0_) > 0.998 for *d* = greatest *Pr*(*Max*)	Success Criteria	No
Max *Pr*(*θ*_*d*_−*θ*_0_<−1) < 0.1	Futility Criteria	Yes

*θ*_*d*_ = mean response for any arm *d*, CrI = Credible Interval.

A pairwise comparison between treatment arms was conducted by estimating posterior probabilities of superiority to assess the relative performance of each route against the others. The posterior probability that Plaza/Control was faster than State Line was high, at 0.99. Additionally, the probability that Rainbow was better than State Line was 0.98, suggesting that Rainbow is the superior route compared to State Line. On the other hand, the probability that Plaza/Control was faster than Rainbow was 0.84. Thus, we concluded that neither route outperformed Plaza/Control.

A sensitivity analysis was conducted where the drive with a protocol violation was excluded. The analysis showed similar findings to our original analysis. More details on the analysis and programming, along with the study timeline progression, response and subject allocation, pairwise comparison can be found in the supplement materials.

## Evaluation of student learning

An instructor can create a way to evaluate students’ learning in the middle or at the end of the course. For example, during our DRIVE trial, an evaluation of the design and implementation was done using Ideaboardz [44] led by the fifth author of the paper. Ideaboardz is a real-time online collaboration tool for brainstorming and retrospectives, which allows users to create a board and discuss a topic. Using this tool after the first interim analysis, we were able to do a qualitative assessment of the students’ satisfaction to find out what went well in the trial and what we could do to improve it. This led to some changes in how we would design future trials. We created three columns for what went well, what did not go well, and ideas for improvement. As for what went well, everyone agreed that the randomization app always worked. The communication between team members was efficient. Although the trial went well, there were some challenges that the group faced. At the beginning of the trial, we decided to have a blinded trial with only the data management team and the protocol team unblinded. However, this led to complications in the coding, analysis, and generation of the next allocation table during the first interim analysis for the statistical team. So, the statisticians were unblinded after the first interim analysis due to practical reasons. Upon reflection, the randomization list for the next interim should have been generated in the mock analysis so that we could catch the blinding issue earlier. This illustrates the importance of using a mock analysis and a clear protocol to identify any issues before the main trial. These helped improve the second interim and final analyses to run smoothly. The sample figure for the Ideaboardz can be found in the supplement.

## Discussion

In this article, we introduce a case study via an open source to guide instructors through designing and implementing a Bayesian adaptive clinical trial in a classroom setting. Biostatisticians and biostatistics graduate students do not always have the opportunity to help design, implement, execute, and analyze a clinical trial from start to finish, so developing ways to enable this experience in the classroom or through small working groups is essential. These opportunities allow students to work together to prepare with the collaboration experience and problem-solving skills. This work focused on providing a framework for instructors to design and conduct adaptive clinical trials by implementing the DRIVE trial, a study designed to find the optimal driving route from the subject’s home to the workplace at the university. Literature around adaptive trial designs and results has grown rapidly throughout the years, but there is a need to present more examples of hands-on collaborative experience to continue to educate and grow this area of expertise [3,5,15].

Though we did not identify a noticeable reduction of driving time when compared to the Control route, this trial allowed the working group to collaborate, discuss, and improve the trial while providing valuable learning experience to the biostatistics students. Student groups proposed their designs to help improve their presentation skills by presenting their part of the project to the group. By discussing pros and cons together as a working group, they experienced how to evaluate alternatives and find compromises. Each group was responsible for certain parts of the trial, which helped to demonstrate the importance of dividing responsibilities while maintaining ownership of the project. The use of real data and Bayesian adaptive features enhanced the engagement and understanding of statistical concepts. The mock trial ensured smooth execution of the main trial of the study. Challenges included blinding complications, which caused coding error and operational complexities. This single subject design may be feasible, but it limited generalizability and engagement for some students who are not familiar with the computing context. Again, coordinating the interim analyses and ensuring consistency may require additional instructor supervision.

Throughout the trial, students were engaged in active learning. From simulating data to using a randomized app, they had effective practical insights in developing tools and deep engagement with statistical concepts [45,46]. They gained practical experience while working in a collaborative group which is needed for their future leadership [47–49]. We hope the DRIVE trial framework encourages other instructors to develop and share more resources like this in the field of adaptive clinical trial design that develop active learning and students’ engagement.

## Limitations

In addition to the motivation for a case study, it is important to formally describe the limitations of the case study, as it provides important context for the educator or learner. While this project template could be extended to other research groups and biostatistics students, we recognize that the specific study design utilized is very personalized. Although the study results are not generalizable, the process of collaborating while designing, implementing, and analyzing a clinical trial can be generalized to answer many different types of questions. Again, because the primary endpoint is drive time, the post-disclosure bias of a route influencing the outcome is minimal. This limitation was discussed with the students so they can understand the challenges one can face while maintaining blinding integrity in clinical trials. The study is best suited for a small class or a workshop. The use of software like FACTS limits accessibility; however, full replicated R code is available for instructors in the supplements to do the design and analyses of their study.

## Recommendations and future work

The DRIVE trial serves as an easy way for any instructor or educator to design and implement a study of interest. However, we recommend keeping the module structure and group-based approach, but in a smaller-scale version, changing the scientific question to better suit their study of interest. For example, the instructor can start with one or two adaptive design elements and look at optimal long-lasting flavor of chewing gum [50], optimal treatment to improve sleep, coffee intake, or other types of research questions to adapt the template on their own. They can incorporate brief sessions on each component to help students engage more with the trial. For larger classes, the focus should be only on the simulated data to manage flexibility during the course or workshop time. If any learner is interested in other types of designs such as designs that allow parametric structures or a normal dynamic linear model, different code will be needed, and a more advanced class could create that. An instructor can challenge the students to do it if they have enough time to do so. We also recommend always including a mock trial before starting a main trial to catch any issues early. Instructors can also adapt for online delivery by using the collaborative tools available. The days of the week were not accounted for in remodeling, which any instructors may be interested in working on in the future. The design could be done by time of day, days of the week, weather, car type etc. Again, SD plays a key role in clinical trials, and its uncertainty could be added using prior information. Instructors may include such priors for further study.

## Supporting information

10.1017/cts.2026.10796.sm001 Alam et al. supplementary material Alam et al. supplementary material

## Supplementary material

The supplementary material for this article can be found at https://doi.org/10.1017/cts.2026.10796.

## Table 1. Long description

A table with two columns and seven rows. The first column is labeled Components and the second column is labeled Purpose. Row 1: Components, 1. Proposed idea; Purpose, Define scientific question, treatment arms, and primary endpoint. Row 2: Components, 2. Design selection; Purpose, Compare different design ideas through simulation and select optimal design. Row 3: Components, 3. Protocol; Purpose, Translate the selected design into a formal trial protocol. Row 4: Components, 4. SAP; Purpose, Defining the model, interim analysis rules, and decision thresholds. Row 5: Components, 5. Data management plan and determination of non-human subjects; Purpose, Details of how data were collected, stored, and protected. Row 6: Components, 6. REDCAP form; Purpose, Details on the RedCap form used for the trial to build the data collection instrument. Row 7: Components, 7. R code; Purpose, Implement the analysis and interpret the results.

Navigate back to Table 1..

## Table 2. Long description

A table titled ‘Drive time (minutes)’ compares drive times across three scenarios for three different routes: Plaza (control), Rainbow, and State line. The table has three rows labeled Null, Expected, and One best, and three columns labeled Plaza (control), Rainbow, and State line. Each cell contains the drive time in minutes for the corresponding scenario and route. Row 1: Null, Plaza (control) 15, Rainbow 15, State line 15. Row 2: Expected, Plaza (control) 15, Rainbow 13, State line 13. Row 3: One best, Plaza (control) 15, Rainbow 13, State line 14.

Navigate back to Table 2..

## Table 3. Long description

The table compares five different study designs based on various criteria. It has six columns: Design type, Number of interims at N drives, Early stopping criteria, Final criteria, Scenario effect sizes, Mean N drives, and Type I error rate or power. The table has five rows for each design type: Fixed, RAR lower interims, RAR arm dropping, RAR higher interims, and RAR no fixed allocation to Control. Each row provides specific details for each design type. For example, the Fixed design has 0 interims, no early stopping criteria, and success as the final criteria. The RAR lower interims design has 3 interims at drives 8, 16, and 24, with futility and success as early stopping criteria, and success as the final criteria. The table also includes data on the mean number of drives and type I error rate or power for different scenarios (null, expected, best).

Navigate back to Table 3..

## Table 4. Long description

The table compares factors affecting drive time across three routes: Plaza (control), State line, and Rainbow. It includes data on weather conditions, car type, construction, start time, and days of the week. The table has 18 rows and 5 columns. Column headers are Route randomized, n (percent), Plaza (control), State line, and Rainbow. Row labels include Weather, Car type, Construction, Start time (hour), and Days of Week. Weather conditions are categorized as Clear, Icy, and Wet. Car type is categorized as GMC. Start time is categorized as 7-8 AM, 8-9 AM, and After 9 AM. Days of Week are categorized as Monday, Tuesday, Wednesday, Thursday, and Friday. Row 1: Weather, Clear, Plaza (control), 2 (33 percent), State line, 0 (0 percent), Rainbow, 7 (88 percent). Row 2: Weather, Icy, Plaza (control), 3 (50 percent), State line, 2 (100 percent), Rainbow, 1 (13 percent). Row 3: Weather, Wet, Plaza (control), 1 (17 percent), State line, 0 (0 percent), Rainbow, 0 (0 percent). Row 4: Car type, GMC, Plaza (control), 6 (100 percent), State line, 2 (100 percent), Rainbow, 8 (100 percent). Row 5: Construction, Plaza (control), 0 (0 percent), State line, 0 (0 percent), Rainbow, 0 (0 percent). Row 6: Start time (hour), 7-8 AM, Plaza (control), 5 (83 percent), State line, 2 (100 percent), Rainbow, 4 (50 percent). Row 7: Start time (hour), 8-9 AM, Plaza (control), 0 (0 percent), State line, 0 (0 percent), Rainbow, 4 (50 percent). Row 8: Start time (hour), After 9 AM, Plaza (control), 1 (17 percent), State line, 0 (0 percent), Rainbow, 0 (0 percent). Row 9: Days of Week, Monday, Plaza (control), 1 (17 percent), State line, 0 (0 percent), Rainbow, 2 (25 percent). Row 10: Days of Week, Tuesday, Plaza (control), 1 (17 percent), State line, 1 (50 percent), Rainbow, 2 (25 percent). Row 11: Days of Week, Wednesday, Plaza (control), 1 (17 percent), State line, 1 (50 percent), Rainbow, 1 (13 percent). Row 12: Days of Week, Thursday, Plaza (control), 1 (17 percent), State line, 0 (0 percent), Rainbow, 2 (25 percent). Row 13: Days of Week, Friday, Plaza (control), 2 (33 percent), State line, 0 (0 percent), Rainbow, 1 (13 percent).

Navigate back to Table 4..

## Table 5. Long description

A table comparing drive times for different routes. The table has 12 rows and 3 columns. The columns are labeled Parameter, Description, and Estimate (95% CrI). The rows provide details on the number of drives, mean times for different routes, standard deviation, mean differences, and probabilities. The first row indicates the number of drives is 16. The mean times for Plaza (Control), Rainbow, and State Line are 11.4, 12.0, and 14.0 minutes respectively. The standard deviation for drive time is 1.2. The mean differences between Rainbow and Plaza, and State Line and Plaza are 0.6 and 2.6 minutes respectively. The probability of Rainbow being the best route is 0.98, while for State Line it is 0.02. The probability of being better than Plaza (Control) for the best route is 0.16. The maximum probability of being better than Plaza (Control) by one minute is 0.01. The success criteria for the greatest probability is not met, while the futility criteria is met.

Navigate back to Table 5..
