# Supplementary material for: A case study in teaching and learning the design, conduction, and analysis of Bayesian adaptive trials through an application
Source: J Clin Transl Sci. 2026 Jul 14;10(1):e134. doi: 10.1017/cts.2026.10796 (PMC13430487; doi:10.1017/cts.2026.10796)

**A Case Study in Teaching and Learning the Design, Conduction, and Analysis of Bayesian Adaptive Trials through an Application**

**Supplementary Materials**

The support materials below include two figures and one table. The detailed case study can be found on GitHub through the link: <https://github.com/BayesPhase/DRIVE>

**Appendix A1.** Study Timeline Progression


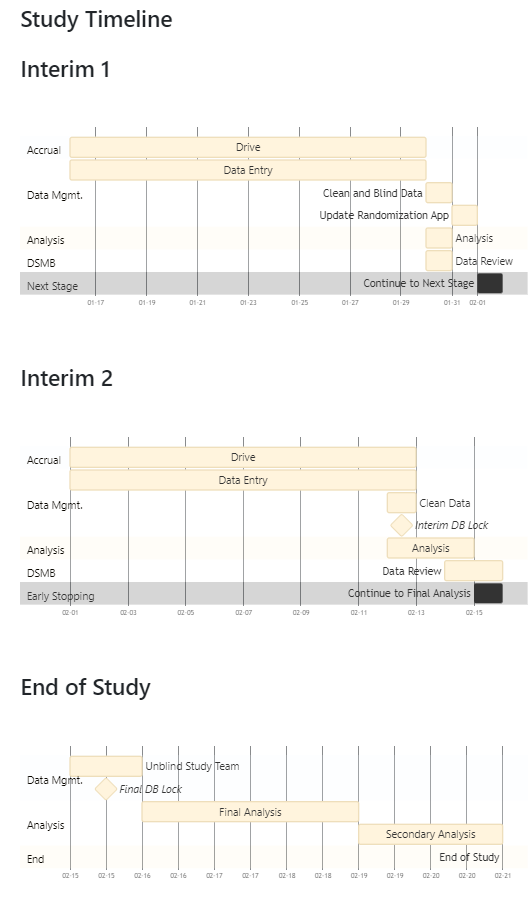


**A2.** Simulation Results for the Chosen Design

| **Scenario** | **Expected Number of Drives** | **Expected Allocation** | | | **Pr(Success)** | **Pr(Futility)** | **Expected Duration in Weeks** |
| --- | --- | --- | --- | --- | --- | --- | --- |
|  |  | **Plaza (Control)** | **Rainbow** | **State Line** |  |  |  |
| **Null (No Effect)** | 23.6 | 7.9 | 7.9 | 7.8 | 0.024 | 0.592 | 4.6 |
| **Expected** | 23.6 | 7.9 | 7.7 | 8.0 | 0.980 | 0.002 | 4.6 |
| **One Best** | 25.1 | 8.3 | 11.6 | 5.3 | 0.944 | 0.006 | 4.9 |

**A3.** First Interim Analysis

| **Parameter** | **Description** | **Estimate  (95% CrI)** |
| --- | --- | --- |
| $\theta_{0}$ | Mean time for Plaza (Control) | 11.7 (10.5, 13.0) |
| $\theta_{1}$ | Mean time for Rainbow | 11.9 (10.3, 13.7) |
| $\theta_{2}$ | Mean time for State Line | 14.0 (12.3, 15.7) |
| $\sigma$ | Standard deviation for drive time | 1.3 (0.6, 2.4) |
| $\theta_{1}-\theta_{0}$ | Mean difference between Rainbow and Plaza (Control) | 0.2 (-1.9, 2.4) |
| $\theta_{2}-\theta_{0}$ | Mean difference between State Line and Plaza (Control) | 2.3 (0.1, 4.4) |
| $Pr(Max) for Rainbow$ | Probability of Rainbow being the best route | 0.95 |
| $Pr\left( Max \right) for State Line$ | Probability of State Line being the best route | 0.05 |
| $Pr\left( \theta_{d}<\theta_{0} \right)$  $\text{for }d=\text{ greatest }Pr\left( Max \right)$ | Probability of being better than Plaza (Control) for the best route | 0.4 |
| $\text{Max }Pr\left( \theta_{d}-\theta_{0}<-1 \right)$ | Maximum probability of being better than Plaza (Control) by one minute | 0.1 |
| $Pr\left( \theta_{d}<\theta_{0} \right)>0.998\text{ }$  $\text{for }d=\text{ greatest }Pr\left( Max \right)$ | Success Criteria | No |
| $\text{Max }Pr\left( \theta_{d}-\theta_{0}<-1 \right)<0.1$ | Futility Criteria | No |
| $\theta_{d}$ is the mean response for any arm *d.* | | |

**A4.** Sensitivity Analysis Results

| **Parameter** | **Description** | **Estimate**  **(95% CrI)** |
| --- | --- | --- |
| N | Number of drives | 15 |
| θ_0_ | Mean time for Plaza (Control) | 11.5 (10.4, 12.4) |
| θ_1_ | Mean time for Rainbow | 12.0 (11.1, 12.9) |
| θ_2_ | Mean time for State Line | 14.0 (12.3, 15.7) |
| σ | Standard deviation for drive time | 1.2 (0.8, 1.8) |
| θ_1_ - θ_0_ | Mean difference between Rainbow and Plaza (Control) | 0.5 (-0.7, 2.0) |
| θ_2_ - θ_0_ | Mean difference between State Line and Plaza (Control) | 2.4 (0.6, 4.6) |
| Pr(Max) for Rainbow | Probability of Rainbow being the best route | 0.98 |
| Pr(Max) for Stateline | Probability of State Line being the best route | 0.02 |
| Pr(θ_d_<θ_0_ ) for  d = greatest Pr(Max) | Probability of being better than Plaza (Control) for the best route | 0.25 |
| Max Pr(θ_d_ - θ_0_ < -1) | Maximum probability of being better than Plaza (Control) by one minute | 0.02 |
| Pr(θ_d_<θ_0_ ) > 0.998 for  d = greatest Pr(Max) | Success Criteria | No |
| Max Pr(θ_d_-θ_0_ < -1) < 0.1 | Futility Criteria | Yes |
| $\theta_{d}$ is the mean response for any arm *d.* | | |

**A5.** Response and Subject Allocation


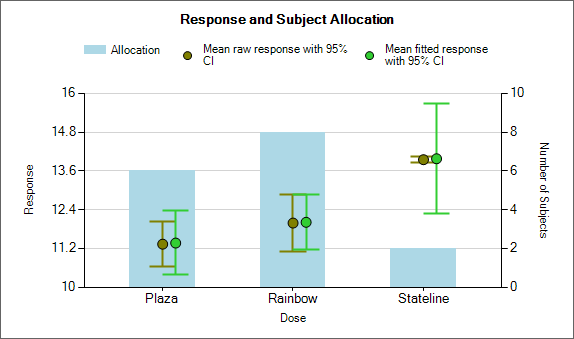

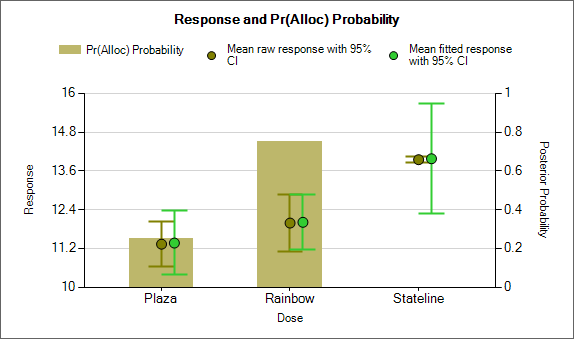


**A6**. Pairwise Comparison

| **Parameter** | **Description** | **Estimate (95% CrI)** |
| --- | --- | --- |
| $\theta_{0}$ | Mean time for Plaza (Control) | 11.4 (10.4, 12.4) |
| $\theta_{1}$ | Mean time for Rainbow | 12.0 (11.1, 12.9) |
| $\theta_{2}$ | Mean time for State Line | 14.0 (12.3, 15.7) |
| $\sigma$ | Standard deviation for drive time | 1.2 (0.8, 1.8) |
| $Pr\left( \theta_{0}<\theta_{1} \right)$ | Probability of Plaza (Control) being better than Rainbow | 0.84 |
| $Pr\left( \theta_{0}<\theta_{2} \right)$ | Probability of Plaza (Control) being better than State Line | 0.99 |
| $Pr\left( \theta_{1}<\theta_{2} \right)$ | Probability of Rainbow being better than State Line | 0.98 |

**A7**. Ideaboardz tool to discuss students’ satisfaction in the trial


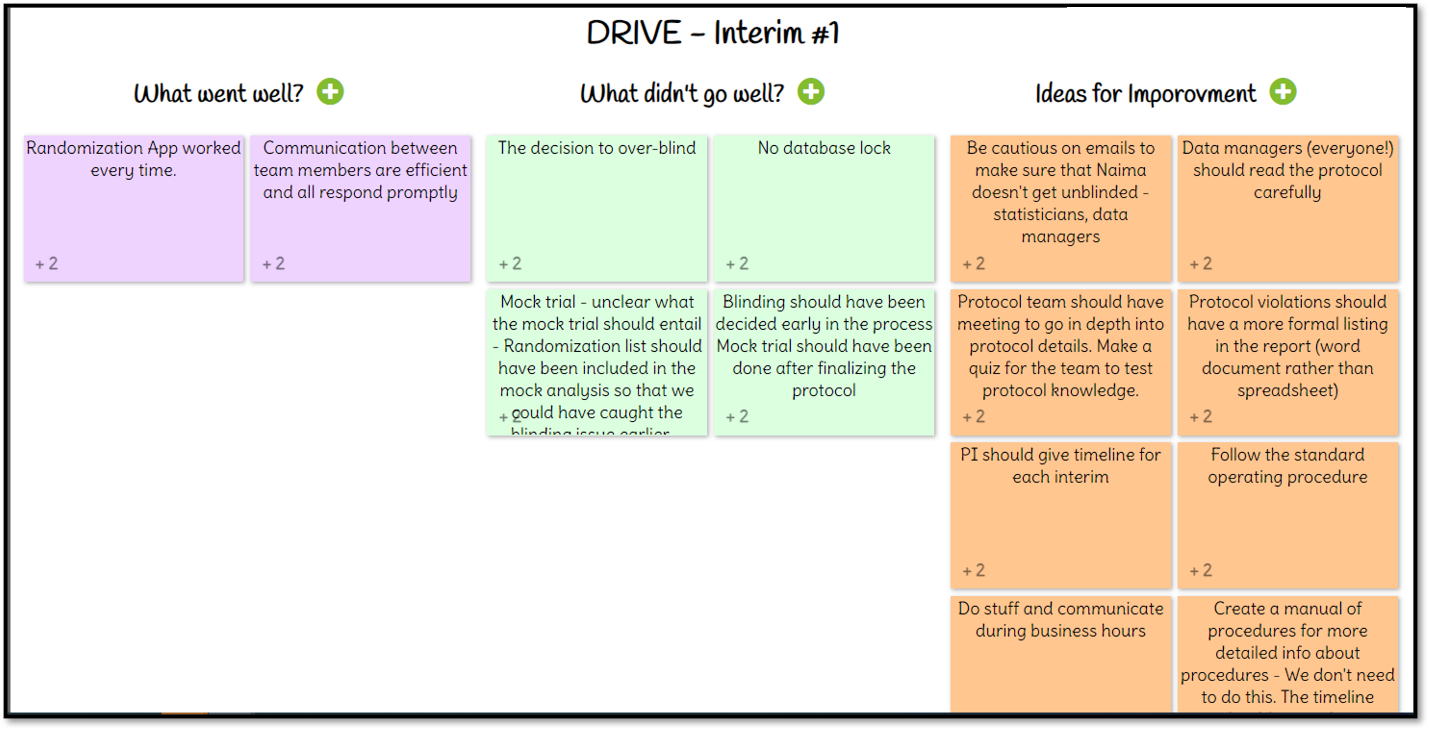

Supplement: Alam et al. supplementary material [file S2059866126107961sup001.docx]
